# Comment on “Efficacy of high energy, focused ESWT in treatment of lumbar facet joint pain - a randomized sham-controlled study”

**DOI:** 10.1097/JS9.0000000000003276

**Published:** 2025-08-26

**Authors:** Yuxi Luo, Qin Luo, Hairong Ding, Yiran Yin

**Affiliations:** a Department of Orthopedic, Changning County Hospital of Traditional Chinese Medicine, Sichan, China; b Department of Rehabilitation Medicine, Changning County Hospital of Traditional Chinese Medicine, Sichan, China; c Department of Orthopedics, the Affiliated Hospital of Southwest Medical University, Luzhou, Sichuan Province, China; d Sichuan Provincial Laboratory of Orthopedic Engineering, Luzhou, Sichuan Province, China

*Dear Editor,*

The randomized sham-controlled trial by Nedelka *et al* provides valuable evidence supporting high-energy focused ESWT as a non-invasive treatment for chronic lumbar facet joint (FJ) pain^[1]^. The reported 64.4% reduction in VAS scores, 42.3% decrease in Oswestry disability index (ODI), and resolution of bone marrow edema (BME) in 58.8% of treated patients at 12 months are impressive outcomes. The study’s strengths include its rigorous sham-control design, long-term follow-up, utilization of diagnostic medial branch blocks (MBB) for patient selection, and incorporation of MRI biomarkers. However, we identified several aspects requiring further discussion to fully contextualize the results and guide future research.

1. The study applied strict exclusion criteria, notably excluding patients with clinical or electrophysiological signs of radiculopathy, disc herniation, nerve root compression, or metabolic neuropathies (e.g., diabetes). While this approach enhances diagnostic specificity for isolated facet joint (FJ) pain, it may limit the generalizability of the findings. In clinical practice, FJ pain often coexists with degenerative disc disease, mild foraminal stenosis, or subclinical neuropathic components, even in the absence of overt radiculopathy^[2,3]^. The significant improvement in PainDETECT scores within the ESWT group (*n* = 27) strongly suggests the presence of a neuropathic component in a subset of patients primarily diagnosed with FJ pain. These exclusion criteria may have inadvertently excluded patients who represent this common clinical overlap. Future studies should consider subgroup analyses or stratified randomization based on the presence or absence of neuropathic features—using validated tools such as the PD-Q or DN4—even in the absence of frank radiculopathy, to determine whether ESWT efficacy varies across the broader FJ pain spectrum.
2. The authors used ultrasound guidance to identify FJ orientation and determine the applicator angle, which is commendable. However, the description lacks sufficient detail on how consistent positioning was maintained across the five weekly sessions. Factors such as inter-operator variability, minor changes in patient posture between sessions, and soft tissue movement under the applicator could influence the accuracy of shockwave delivery to the medial branch and facet joint structures, especially given the small focal zone. The study reports delivering 600 shocks per segment (e.g., L3/L4 and L4/L5 for L4/L5 FJ pain), targeting medial branch regions. It remains unclear whether the energy delivery was directed specifically at the anatomical location of the medial branches—requiring precise knowledge of their variable course relative to bony landmarks^[4]^—or broadly applied over the facet joint and adjacent pars region. Clarification regarding the exact anatomical target (e.g., joint capsule, medial branch, or subchondral bone) and standardization methods (e.g., image verification or positioning jigs) would enhance reproducibility. The favorable outcomes observed in high-BMI patients (>28) suggest effective tissue penetration; however, depth-specific dosimetry data would provide additional insight.
3. The sham procedure using an air-filled polyurethane interface is a well-established method. Nevertheless, supplementary data (available online) indicate that 95% of patients in the active group correctly identified their treatment allocation, compared to 75% in the sham group. This raises concerns about the integrity of blinding, particularly for patients in the active treatment arm. Given the distinctive sensation associated with high-energy ESWT (0.35 mJ/mm^2^), this unblinding may have influenced subjective patient-reported outcomes (PROs), such as the VAS and ODI, potentially inflating the perceived treatment effect. Reporting the correlation between treatment guessing and outcome magnitude, or analyzing outcomes among only those patients who remained blinded, could help assess this potential bias. While objective MRI measures (e.g., BME resolution) are less prone to bias, the primary endpoints in this study were PROs.
4. The observed resolution of bone marrow edema (BME), particularly Modic changes, in 58.8% of treated patients is both novel and compelling, suggesting a potential biological effect on subchondral inflammation. However, Modic Type I changes are known to fluctuate spontaneously^[5]^. Although the absence of change in the sham group is reassuring, the study lacked a natural history control arm. It remains unclear whether patients with BME resolution had specific baseline characteristics (e.g., shorter symptom duration, specific Modic type, or lower BMI) compared to non-responders. Moreover, the relative contributions of BME resolution versus neuromodulatory effects—suggested by improvements in PD-Q scores—are not fully delineated. Future mechanistic studies incorporating serial imaging and molecular biomarkers (e.g., cytokines, neurotrophins) are warranted to clarify these effects.
5. The 12-month follow-up results are highly promising. However, chronic spinal pain conditions like FJ syndrome often follow a relapsing-remitting course, making longer-term data (e.g., at 24 months) essential for confirming the durability of ESWT’s effects. Although the reported outcomes compare favorably to the 50–60% pain relief typically seen with radiofrequency medial branch neurotomy (RF-MBN), direct comparative trials are needed. A randomized trial comparing ESWT and RF-MBN in similar patient populations would be valuable to determine relative efficacy, safety, cost-effectiveness, and patient preference. While the present study confirms ESWT’s superiority over sham, its comparative role against the current invasive standard requires further investigation through head-to-head trials.
6. Nedelka *et al* provide compelling evidence that high-energy focused extracorporeal shockwave therapy (ESWT) is a safe and effective non-invasive treatment for chronic lumbar facet joint pain, demonstrating sustained benefits in pain relief, functional improvement, reduction of neuropathic symptoms, and resolution of structural inflammation (bone marrow edema) at 12 months. This study represents a significant advancement in the field of non-invasive spinal pain management. Future research addressing the aforementioned issues—patient selection, targeting precision, blinding integrity, mechanisms underlying BME resolution, and long-term treatment durability—will help further define the role of ESWT within clinical practice and support the broader implementation of these promising findings. We commend the authors for this valuable contribution to the field.

This article complies with TITAN Guidelines 2025^[6]^.

## Data Availability

Not applicable.
